# Effects on the Titanium Implant Surface by Different Hygiene Instrumentations: A Narrative Review

**DOI:** 10.7759/cureus.30884

**Published:** 2022-10-30

**Authors:** Wong Yen Nee, Raja Azman Raja Awang, Akram Hassan

**Affiliations:** 1 School of Dental Sciences, Universiti Sains Malaysia, Kelantan, MYS

**Keywords:** topographical changes, surface roughness, hygiene instrument, surface effect, dental titanium implant

## Abstract

Peri-implant disease is usually caused by the accumulation of dental biofilm around the implant, and this biofilm can irradiate the gingiva tissue, which leads to inflammation and, more severely, to a deterioration of the bone structure. There is a concern regarding the removal of biofilm from the implant surface by using different hygiene instruments. Some hygiene instruments may have some effect on the dental implant surface, resulting in roughening or damage to the implant surfaces. This study reviewed the effects of titanium implant surfaces on different hygiene instruments. A literature search was conducted from PubMed, ScienceDirect, and Scopus databases for articles published from 1992 to 2021. A total of 19 full-text papers with keywords of interest that met all the eligibility criteria were selected. Surface roughness was evaluated with a scanning electron microscope and also using a profilometer, laser scanning, scanning probe, and atomic force microscopes. A metal curette produced a roughened surface on the titanium implant, but a plastic curette did not alter the surface. Instrumentation with rubber cups left the surface unchanged and appeared to smoothen the surface, whereas the air-powder abrasive instrumentation altered the surface with the presence of micro pits and pores. A conventional metal ultrasonic scaler showed significant surface topographical changes and scratches on both titanium surfaces, as a diode laser, light-emitting diode (LED), and laser treatment did not show any alteration on the rough and smooth titanium surfaces. Thus, a non-metallic instrument such as a plastic curette, rubber cups, and novel technology including diode laser, LED, and laser treatment is appropriate and can be used for debridement on smooth and machined titanium implant surfaces as well as sandblasted and acid-etched (SLA), titanium plasma-sprayed (TPS), and resorbable blasted media (RBM) surfaces. The use of metallic instruments should be avoided, and it is not recommended.

## Introduction and background

The loss of teeth, either partial or full edentulism, has led to the evolution and development of dental implants that have been manufactured using different materials. A dental implant is a metallic component that is surgically placed inside the jaw and serves as a root for the missing tooth or teeth. Titanium has become a material of choice for the manufacture of different components over the last three decades [1].

The success of the first titanium dental implants in the 20th century led to a greater improvement in the tooth replacement technique [2]. Currently, titanium implants are widely used, and they play a major role in restoring the lost tooth and helping in maintaining the aesthetic and function of the tooth inside the patient’s oral cavity [3]. A titanium implant is used to replace lost teeth because of its high level of biocompatibility and it can last longer due to its high resistance to corrosion [4].

However, failure to take care of titanium implants will lead to some peri-implant diseases such as peri-implant mucositis and peri-implantitis. According to the American Academy of Periodontology (AAP), peri-implant disease is an inflammatory condition around the dental implant that affects the soft tissue, particularly the gingiva, and hard tissue such as bone. Peri-implant disease is usually caused by an accumulation of dental biofilm around the implant, and this biofilm can induce inflammation of the gingiva tissue and more severely lead to a deterioration of the bone structure. Thus, it is important to clean the surface of dental implants with home-care techniques daily and regular follow-up and maintenance appointments.

There is a concern regarding the removal of bacterial plaque or biofilm from the implant surface by using different hygiene instruments in clinical settings. Some hygiene instruments might cause some effect on the dental implant surface which causes roughening or damaging of the implant surfaces [5]. The damage to the implant surfaces might cause more plaque accumulation and retention, which would lead to a major problem if left untreated.

There are various hygiene instruments proposed in the previous studies for the cleaning of the titanium implant. These hygienic instruments are more widely tested and are more commonly found. Different instruments produce different surface effects on the titanium implant. It is important that we should use the most suitable and appropriate hygiene instrument to clean titanium implants so that we can maintain the longevity of titanium implants. Therefore, this study aimed to conduct a review of the effects on titanium implant surfaces of different hygiene instruments and identify the most appropriate hygiene instrument that can be used to clean titanium dental implants.

## Review

Materials and method

Study Design and Search Strategy

This was a narrative overview of the literature from various previous research that was retrieved from searches of computerized databases, manual searches, and authoritative texts. The information was searched using the following keywords: "dental titanium implant," "surface effect," and "hygiene instrument." All these keywords were used to find some articles and journals that were reviewed for this research.

Inclusive Criteria and Exclusive Criteria

This study included articles that had keywords of interest, were published in English and were available from trusted data sources such as PubMed, ScienceDirect, and Scopus databases for articles published from 1992 to 2021 that met all the eligibility criteria. Studies that were published from Wikipedia or unknown sources, published in other languages, and not available in full text, and case reports were excluded.

Quality Assessment

The quality assessment tool for this research was taken from Appendix D of Hawker et al. [6]. This assessment contains nine questions that could be answered as "good (4 points)," "fair (3 points)," "poor (2 points)," or "very poor (1 point)." The total points were calculated and the overall quality grades were used following the definitions: high quality (A), 30-36 points; medium quality (B), 24-29 points; low quality (C), 9-24 points.

Results

PubMed, ScienceDirect, and Scopus searches identified 1687, 568, and 1060 papers, respectively. The initial screening of titles and abstracts identified 20 full-text papers. After full-text reading of the articles, one study was excluded because the discussion of the results was not based on a clear evidenced explanation and no data were provided on the surface effect after instrumentation [7]. Finally, a total of 19 full-text papers were processed for data analysis. Most studies included were in-vitro except one, which was carried out in-vivo with 14 patients; 4 males and 10 females participated in that study [8]. A total of 21 titanium implants were placed in the mandibular molar region, and the surface characteristics of the titanium was evaluated using a scanning electron microscope.

For 18 out of 19 studies, surface changes caused by instrumentation were assessed using a scanning electron microscope (SEM). Seven of them were also measured with either a profilometer, scanning probe microscope, laser scanning microscope, or atomic force microscope. Only a study by Biazussi et al. used the only profilometer to access the surface roughness [9]. Most studies looked at the impact of instrumentation on smooth implant surfaces, whereas 10 studies evaluated the impact of instrumentation on rough surfaces such as sandblasted and acid-etched (SLA), titanium plasma-sprayed (TPS), double acid-etched, and resorbable blasted media (RBM) surfaces.

In the SEM analysis, photomicrographs were taken after each instrumentation. They were either used to compare with the control group or used to compare with the pre-treatment implant surfaces. The surface alteration could be visualized using SEM. Mengel et al. evaluated the surface roughness and the loss of implant substance by comparing with the control group using the Modified Roughness Loss of Tooth Substance Index whereby it was assessed in 4 grades: none, slight, moderate, and pronounced roughened [10]. None (score 0) is when the implant surface shows no visible marks and there is no loss of implant surfaces; slight roughened (score 1) is when there is a minimal amount of work traces and minimal loss of implant surfaces; moderate roughened (score 2) is when the marks are visible and there is moderate substance removal; pronounced roughened (score 3) is when there are pronounced work traces and high substance removal of the implant surfaces. There were also a number of studies that used three-dimensional reconstruction of the SEM images, which provided a better image of the surface alteration after instrumentation [2,11].

For surface roughness analysis, a number of studies used a profilometer and compared the data with the control group [9,12,13] or compared between pre-and post-treatment [14,15]. Other studies used a scanning probe microscope [16], a laser scanning microscope [2], or an atomic force microscope [17] to evaluate the surface roughness. The P-value is an important indicator that can help to detect the significance of the difference between pre-and post-treatment. P < 0.05 shows that the difference between the pre and post is significant, which indicates that the surface alteration is significant and vice versa.

Based on all the included studies, the hygiene instruments tested were metal curette, titanium curette, plastic curette, rubber cups, air-powder abrasive, and an ultrasonic scaler with different types of tips, diode laser, light-emitting diode (LED), and laser.

Metal curettes produced a roughened surface on the titanium implant [10,18]. Duarte et al. stated that the titanium surface was found to be roughened by the metal curette and many scratches were produced on the smooth surface, whereas, on the rough titanium surface, it appeared to smoothen the surface and the ridges were flattened [14]. Besides that, titanium curettes produced pronounced marks on the implant surfaces on both smooth and rough surfaces [10]. On smooth titanium surfaces, plastic curettes did not alter the titanium surface and there were no visible changes noted on the surface [10,14,18]. Similarly, on SLA rough surfaces, plastic curettes also did not alter the titanium surfaces [14]. However, Mengel et al. stated that there were visible marks noted on the TPS rough titanium implant surface after instrumentation using a plastic curette [10].

Rubber cups are also one of the instruments used to clean the titanium implant. There were two studies that supported the use of rubber cups because rubber cups left the implant surface unchanged and appeared to smooth the titanium surface and decrease previous machined marks [10,18]. There were also two studies stating that the rubber cup produced irregular surfaces and produced uniform lines of scratches on the titanium surface [5,19].

Although Mengel et al. and Homiak et al. stated that the air-powder abrasive system did not alter the titanium surfaces [10,18], this was opposed by Duarte et al., who stated that air-powder abrasive produced an irregular surface on the smooth titanium implant and flattened the rough edges of the rough surface [14]. This was also supported by Hassan et al. and Bahari et al., who stated that the titanium surface was altered and there was the presence of micro pits and pores in the air-powder abrasive-treated group [5,19]. There was also a study that compared the two different types of abrasive powder and found that air-powder abrasive with amino acid glycine produced less surface roughness compared to air-powder abrasive with sodium bicarbonate on titanium implant surfaces [9].

The ultrasonic scaler is widely used in scaling. Many studies stated that the conventional metal ultrasonic scaler showed remarkable surface topographical changes and scratches either on the smooth, rough, or both titanium implant surfaces [2,8,10-13,20]. There were studies that claimed that ultrasonic with a plastic tip produced a roughened surface on both smooth and rough titanium implant surfaces [2,10]. However, Sahrmann et al. claimed that plastic tips altered the rough titanium implant by flattening the moderately rough surface but did not alter the smooth titanium implant surface [12]. Other than a conventional and plastic ultrasonic scaler, Sinjari et al. and Unursaikhan et al. supported the use of an ultrasonic scaler with a copper alloy silver-plated tip because it showed no marked changes to the titanium surface [11,13]. Carbon and resin-tipped ultrasonic scalers could also be used because they did not alter the smooth and rough titanium implant surface [12]. In the same study, they opposed the use of an ultrasonic scaler with a titanium tip because it showed visible traces and scratches on the smooth titanium implant surface and altered the rough titanium implant surfaces.

A diode laser is a new technology to clean titanium implant surfaces. It was supported by a few studies stating that diode lasers would not alter the surface of the titanium implant surface at certain parameters [16,21,22]. However, at higher energies of 4.5 and 5.5 W, there were surface damages whereby melting of the irradiation site was visible [23]. LED could be used to clean the titanium implants because it did not show any alteration on the rough and smooth titanium implant surface at the tested parameters [21,22]. Lasers such as the erbium-doped yttrium aluminium garnet laser (Er:YAG) produce alteration on both smooth and rough surfaces of titanium implants [14]. In addition, there were also signs of melting and microfractures noted [17]. However, there was a study that claimed that the Er:YAG laser did not produce any alteration on the rough titanium implant surface [22]. Kim et al. and Matsuyama et al. also stated that the Er YAG laser showed no visible alterations to the titanium surface at a lower strength [15,20]. However, at a higher strength, distinct colour changes and morphological changes due to the melting of the titanium surface were noted at the irritation spot. Other lasers, such as the neodymium-doped yttrium aluminium garnet laser (Nd:YAG) showed no evident changes on the titanium implant surface at all parameters [24]. A summary of studies evaluating the effects of different types of hygiene instruments on titanium implant surfaces has been tabulated (Table 1).

**Table 1:** Summary of studies evaluating the effect of different types of hygiene instruments on titanium implant surfaces TPS: titanium plasma-sprayed; SLA: sandblasted and acid-etched; LED: light-emitting diode; Nd:YAG: neodymium-doped yttrium aluminum garnet; Er:YAG: erbium-doped yttrium aluminium garnet

References	Surface roughness analysis	Results
[10]	Scanning electron microscope	Smooth surface: Plastic curette, rubber cups, and air-powder abrasive left the implant surface unchanged. Titanium curette and disposable plastic ultrasonic scaler produced gentle marks on the implant surface. Metal curette, conventional ultrasonic scaler, and plastic ultrasonic scaler produced a roughened surface on the titanium implant surface; rough surface (TPS): apart from rubber cups and air-powder abrasive, all other instruments produced a pronounced mark on the rough titanium implant surface.
[18]	Scanning electron microscope	The titanium surface was found to be roughened by the metal curette. Plastic curette, rubber cup, rubber cup with tin oxide, and air-powder abrasive appeared to smooth the titanium surface and decrease previous machined marks.
[13]	Scanning electron microscope, profilometer	Ultrasonic scaler with copper alloy silver-plated tip and plastic curette showed no marked changes to titanium surface. However, ultrasonic scaler with conventional tip and root planer ultrasonic scaler damaged the titanium surface and produced an irregular surface.
[19]	Scanning electron microscope	Surface was altered and presence of micro pits and pores in the air-powder abrasive treated group whereas rubber cup and pumice showed uniform lines of scratches on the titanium surface.
[5]	Scanning electron microscope	Air-powder abrasive system created a generalized micropores on the titanium implant abutment, whereas rubber cups with pumice powder produced an irregular surface on titanium surface.
[11]	Scanning electron microscope	Ultrasonic scaler with conventional steel tip showed irregularities and presence of groove on the titanium surface whereas innovative copper alloy silver-plated tip had minimal changes on the titanium surface.
[2]	Scanning electron microscope, laser scanning microscope	Machined and rough surface: Ultrasonic scaler with metallic tip showed significant surface topographical changes while plastic tip showed mild to moderated alteration on both machined and rough surface titanium surface.
[8]	Scanning electron microscope	Ultrasonic scaler with carbon and plastic tip produced minimal surface damage on the abutment surface than metallic tip.
[12]	Scanning electron microscope, profilometer	Smooth surface: Ultrasonic scaler with resin, carbon and plastic tip did not alter the smooth titanium implant surface while steel and titanium tip showed visible traces and scratches on the smooth titanium implant surface; rough surface (SLA): ultrasonic scaler with steel, titanium, and plastic tip altered the rough titanium implant by flattening the moderately rough surface whereas resin and carbon tip did not alter the rough SLA surface.
[9]	Profilometer	Air-powder abrasive with amino acid glycine produced less surface roughness compared to air powder abrasive with sodium bicarbonate on titanium implant surface. Air-powder abrasive with amino acid glycine had similar surface roughness with the control group.
[16]	Scanning electron microscope, scanning probe microscope	Diode laser did not significantly alter the surface of the rough titanium implant surface at all parameters.
[21]	Scanning electron microscope	Both diode laser and LED did not show any surface alteration, cracks, or damage to the titanium implant compared to control group.
[24]	Scanning electron microscope	Smooth and rough surface: Diode laser and Nd:YAG laser showed no evident changes on the titanium implant surface at all parameters.
[23]	Scanning electron microscope	The rough titanium implant surface did not show any surface damage with laser irradiation at 1.5, 2.5, and 3.5W. However, energies at 4.5 and 5.5W showed surface damages whereby melting of the irradiation site was visible.
[22]	Scanning electron microscope	Laser irradiation with Er:YAG, LED, and diode laser at respective parameters did not show any alteration on the rough titanium implant surface.
[14]	Scanning electron microscope, profilometer	Er:YAG laser produced slight alteration on both smooth and rough surface of titanium implant. Plastic curette showed no visible changes on both smooth and rough surface of titanium implant; smooth surface: Titanium surface was found to be roughened by the metal curette and many scratches were produced on the smooth surface. Air-powder abrasive produced an irregular surface on the smooth titanium implant; rough surface (SLA): metal curettes appeared to smoothen the rough titanium surface by flattening the ridges. Air-powder abrasive also flattened the rough edges of the rough surface.
[20]	Scanning electron microscope	In contrast to ultrasonic scaling, which caused severe surface damage to the titanium surface, the Er:YAG laser treatment effectively removed calculus with no visible alterations to the titanium surface at 30 mJ/pulse and 50 mJ/pulse. However, when the strength increases to 100 and 200 mJ/pulse, distinct colour changes and morphological changes due to melting of the titanium surface were noted at the irritation spot.
[15]	Scanning electron microscope, profilometer	Rough surface (double acid-etched): laser irradiation with 100 mJ/pulse did not alter the rough titanium implant surface. However, laser irradiation with 140 and 180 mJ/pulse increasingly altered the rough titanium implant surface by reducing sharpness of the ridges.
[17]	Scanning electron microscope, atomic force microscope	Laser irradiation at 1 W and 100 mJ/pulse showed minor surface alteration such as microcracks whereas laser irradiation at 4W and 400 mJ/pulse showed remarkable alteration whereby signs of melting and microfractures were visible on the rough titanium surface.

A plastic curette could be used to remove calculus and plaque from implant surfaces supragingivally without causing damage [10,14], whereas a metal curette was not suitable for treating smooth surfaces [14,18]. Hassan et al. and Bahari et al. noticed that rubber cups and pumice were more effective and less invasive on titanium implant surfaces [5,19], and this conclusion was also suggested by Mengel et al. [10]. For the air-powder abrasive instrumentation, Hassan et al. and Bahari et al. concluded that air-powder abrasive was less effective and more invasive on titanium implant surfaces [5,19], but Biazussi et al., Mengel et al., and Duarte et al. suggested that air-powder abrasive was suitable for the debridement [9,10,14].

Copper alloy silver-plated ultrasonic scaler was suitable for peri-implant surface decontamination because of its limited effect on titanium surface as concluded by Sinjari et al. and Unursaikhan et al. [11,13]. Tawse-Smith et al. noted that ultrasonic scalers of metallic and plastic types were not suitable to be used on titanium implant surfaces because they caused compositional changes on the surface [2], as Kawashima et al. and Sahrmann et al. concluded that carbon tip caused the least change on the implant surface [8,12].

In general, diode laser beams might be useful to treat peri-implantitis because they do not damage implant surfaces. It was a successful treatment option to be used as a hygiene instrument on titanium implant surfaces [16,21-24]. The Er:YAG laser could be a novel choice for the debridement of the titanium surface of implant surfaces [14,15,20,22], but it also resulted in changes to the topography of the titanium implant surfaces [17]. A summary of studies identifying the most appropriate hygiene instrument used to clean titanium implants has been tabulated (Table 2).

**Table 2:** Summary of studies identifying the most appropriate hygiene instrument used to clean titanium implant Nd:YAG, neodymium-doped yttrium aluminum garnet; Er:YAG, erbium-doped yttrium aluminium garnet; LED, light-emitting diode; ICG, indocyanine green

References	Hygiene instrument tested	Conclusion
[10]	Titanium curette, metal curette, plastic curette, rubber cups with prophylaxis paste, conventional ultrasonic scaler, air-powder abrasive, conventional disposable plastic ultrasonic scaler	Rubber cup, plastic curette and air-powder abrasive can be used to remove calculus and plaque from implant surfaces supragingivally without causing damage
[18]	Metal curettes, plastic curettes, rubber cup, rubber cup with tin oxide, air-powder abrasive with sodium bicarbonate powder	Metal scaler was seen to roughen the titanium implant surface. Further studies should be done for conclusive effect
[13]	Ultrasonic scaler (copper alloy silver-plated and conventional), root planer ultrasonic scaler, plastic curette	Copper alloy silver plated ultrasonic scaler is suitable for peri-implant surface decontamination because of its limited effect on titanium surface
[19]	Air-powder abrasive with amino acid glycine powder, rubber cup with pumice powder	Rubber cup with pumice is more effective and less invasive on titanium implant surface compared to air-powder abrasive system
[5]	Air-powder abrasive with amino acid glycine powder, rubber cup with pumice powder	Rubber cup with pumice shows less prominent machined lines on titanium implant than air-powder abrasive system
[11]	Ultrasonic scaler (conventional steel and innovative copper alloy silver-plated)	Ultrasonic scaler with innovative copper alloy silver-plated tip is more suitable for peri-implant surface decontamination due to lesser implant surface alterations
[2]	Ultrasonic scaler (metallic and plastic)	Ultrasonic scaler of both types are not suitable to be used on titanium implant surface because they cause compositional changes on the surface
[8]	Ultrasonic scaler (carbon, plastic, and metallic)	Ultrasonic scaler with non-metal tips (carbon and plastic tip) are suitable to use on titanium implant surface
[12]	Ultrasonic scaler (steel, polyether ether ketone plastic, titanium, carbon and resin)	Resin and carbon tips cause least change on the implant surface
[9]	Air-powder abrasive (sodium bicarbonate and amino acid glycine)	Air-powder abrasive with amino acid glycine produces less effect on the titanium implant surface
[16]	Diode laser (940 nm and 980 nm at 1.0, 2.0, and 3.0 W)	Diode laser with 1.0, 2.0, and 3.0 W output can decontaminate the SLA titanium surface without damage
[21]	Diode laser (660 nm, 100 mW) with phenothiazine chloride dye, LED (660 nm)	Diode laser is a successful treatment option to be used as a hygiene instrument on titanium implant surfaces
[24]	Diode laser (980 nm at 2.5 and 3.0W), Nd:YAG laser (1064 nm at 2.5W and 3.0 W)	The 980 nm diode and 1064nm Nd:YAG lasers are effective in bacterial decontamination without causing surface change on the titanium implants
[23]	Diode laser (810 nm at 1.5, 2.5, 3.5, 4.5, and 5.5W)	Diode laser beam of up to 3.5W might be useful to treat peri-implantitis because it does not damage implant surfaces
[22]	Er:YAG laser (1W, 100 mJ/pulse), LED (630 nm) with toluidine blue, Diode laser (810 nm, 300 mW) with ICG-based photosensitizer	Er:YAG, LED and diode laser can be used on rough titanium implant surfaces because they do not alter the titanium implant surfaces
[14]	Er:YAG laser (120 mJ/pulse, 10 Hz), plastic curette, metal curette, air-powder abrasive with sodium bicarbonate	Metal curette is not suitable in treating smooth surface whereas all other instrument tested are suitable for the debridement of rough surfaces
[20]	Er:YAG laser (1 W with 30, 50, 100 and 200 mJ/pulse), ultrasonic scaler with universal tip	The Er:YAG laser at 30 and 50 mJ/pulse can be a novel choice for debridement of the titanium surface of implant abutments
[15]	Er:YAG laser (100, 140, and 180 mJ/pulse)	Laser beam less than 100mJ/pulse at 10 Hz for less than 2 minutes can be used to detoxify implant surface without causing surface alteration
[17]	Er:YAG laser (1W, 100 mJ/pulse and 4W, 400 mJ/pulse)	Both protocols of Er:YAG laser irradiation result in changes to the topography of the titanium implant surfaces

Discussion

The long-term success of an implant is highly dependent on the long-term implant maintenance of the soft and hard tissue. Implant maintenance can be done through daily home care techniques and professional oral hygiene care [25]. Everyday home care includes brushing, interproximal cleaning, locally applied chemotherapeutics, and water irrigation, which can be done by the patients themselves. Professional hygiene care is carried out by health care providers such as dentists, which includes scaling and polishing, locally applied chemotherapeutics, and subgingival irrigation [26].

However, the instrumentation on the titanium implant surface may cause surface implant damage over time, resulting in changes in surface topography that may increase bacterial deposition and plaque accumulation [13]. Bacterial infection is one of the main etiological factors that cause implant failure since it causes inflammation around the implant known as peri-implantitis [25]. In order to stop or prevent the progression of the disease, it is crucial to remove plaque and calculus completely from the implant surface using instruments that are light, disposable or sterilizable, easy to use, cost-effective, and most importantly, do not cause any damaging effect on the implant surface [13,26]. Several hygiene instruments such as a metal curette, titanium curette, plastic curette, rubber cups, air-powder abrasive, ultrasonic scaler with different types of tips, diode laser, LED, and laser were proposed. Therefore, in this study, the conventional and novel hygiene instruments were reviewed and compared to identify the most appropriate hygiene instrument used to clean titanium implants.

Metallic and Non-metallic Instruments

Metallic instruments such as stainless-steel curettes and titanium curettes cause the most damage to the titanium implant surface compared to non-metallic instruments. Metallic instruments should not be used as hygiene instruments for titanium implants because they can scratch, roughen, and contaminate the titanium implant surface [10,14], thus making the titanium implant surface more prone to bacterial plaque and calculus accumulation [26]. An ultrasonic scaler with a conventional metal tip is also not appropriate to clean titanium implants because it scratches the titanium implant surface and causes significant surface topographical changes [2,8,10,12]. Unursaikhan et al. claimed that a piezoelectric ultrasonic scaler with a newly developed metallic tip was especially suitable for peri-implant surface decontamination because it showed no significant changes in the titanium surface and had a limited effect on the surface of the titanium implant [13]. Nevertheless, more clinical studies should be carried out to verify the results.

A non-metallic instrument such as a plastic curette can be safely used to clean the titanium implant surface because it shows no visible changes on the titanium implant surface. A plastic scaler is also a choice of hygiene instrument for titanium implants. SEM analysis revealed that the plastic scaler showed no marked changes on the titanium implant and there was some smoothening on the titanium implant [13]. Similarly, other studies also agreed that plastic scalers appeared to smoothen the titanium implant surface and round off the sharp machined grooves [18]. There was also a study that stated that there was no difference in the surface effect for the pre and post-treatment using plastic scalers [27]. Some studies also claimed that plastic instruments may be the choice for the debridement of titanium implant surfaces and produce the least damaging surface effect [7,28].

Ultrasonic Scaler

Ultrasonic scalers with carbon and resin tips can also be used as a hygiene instrument to clean the titanium implant surfaces as they do not cause topographical changes to the titanium implant surface. Sahrmann et al. concluded that resin and carbon tips of ultrasonic scalers caused the least changes to the titanium implant than other types of tips such as plastic, steel, and titanium tips [12]. However, caution must be taken when using these instruments. A short working stroke and light pressure should be used to prevent traumatization to the peri-implant soft tissue [26]. Similarly, a plastic ultrasonic scaler was not recommended to clean the titanium implant surface. Although it caused slight alteration on the titanium implant surfaces, it was more suitable for removing a course deposit rather than calculus and plaque [10].

Air-Powder Abrasive and Rubber Cup

Some studies supported the use of air-powder abrasive and some studies contradicted it. It should be noted that several factors, including water flow, exposure duration, particle size and hardness, air pressure, and nozzle-target distance, may influence the abrasive capability of the systems and, as a result, their effects on titanium surfaces [29]. One study compared the different types of abrasive powder and the results showed that air-powder abrasive with amino acid glycine produced less effect on the titanium implant surface compared to sodium bicarbonate [9]. Hence, more studies should be conducted to include all these factors in order to produce much more reliable results. Besides that, an air powder abrasive system with sodium bicarbonate was believed to cause little or no surface damage to the titanium implant, and it was also the most effective method of cleaning the titanium implant [28]. Likewise, other studies also agreed that an air-powder abrasive system with sodium bicarbonate appeared to smoothen the titanium implant surface and remove the surface debris [18]. On the other hand, a study by Hassan et al. found that titanium implants treated with an air powder abrasive system with glycine showed many prominent machine lines on the titanium implant surface with generalized micro pits and pores [5].

Rubber cups can also be used as hygiene instruments for titanium implants. In one of the studies, it was stated that rubber cups with flour of pumice created a smoother surface than the non-treated titanium implant [27]. This was also agreed upon by other studies stating that there were fewer prominent machine lines produced with the least scratches and pits when treated with rubber cups and pumice [5]. However, the study by Brookshire et al. claimed that other hygiene methods they tested, including rubber cups with tin oxide slurry, either created a significant surface alteration or left residual particles on the surfaces, or both [30].

Diode Laser, LED, and Laser Therapy

Novel hygiene instruments such as diode lasers, LEDs, and laser therapy are recommended for the debridement of the titanium implant surfaces using optimum parameters. Based on Kim et al., it was recommended to use a laser beam of less than 100 mJ/pulse at 10 Hz for less than two minutes so that it could be used to detoxify the implant surface without causing surface alteration [15]. However, certain precautions should be taken, which include the laser output, optimum laser parameter, water irrigation volume, heat generation from laser irradiation, effect on the surrounding tissue, and exposure time [31]. Safety measures should be taken to avoid injuries to the soft and hard tissues. The dentist should also be trained and certified before performing laser treatment.

One of the limitations of this review is that the selection and evaluation of biases in the published article or journal regarding this topic are not known. For instance, it is difficult to predict under which circumstances, excluding studies published in languages other than English may bias this review. Another limitation in this review is the validity of the outcome intraorally. Only one study carried out in-vivo was included [8], and the others were in-vitro studies. It is certain that this is not sufficient to demonstrate the effect of hygiene instruments on titanium implants intraorally. The outcome of the hygiene instruments intraorally was unpredictable because there were many factors to be considered. For instance, some studies in-vitro contaminated and decontaminated the titanium implant surface, but there were differences between these processes in-vitro and intraorally. Clinical trials are needed to correlate these results with in-vivo effects in order to apply these instruments in implant maintenance therapy.

## Conclusions

The metallic instrument should be avoided on titanium implant surfaces. A non-metallic instrument such as a plastic curette, rubber cups, and novel technology including diode laser, LED, and laser treatment is appropriate and can be used on smooth, machined, SLA, TPS, and RBM titanium implant surfaces for debridement. Further clinical studies should be carried out and the clinical impact of these findings should be clarified.

## References

[1] Gubbi P, Wojtisek T (2018). The role of titanium in implant dentistry. Titanium in Medical and Dental Applications.

[2] Tawse-Smith A, Atieh MA, Tompkins G, Duncan WJ, Reid MR, Stirling CH (2016). The effect of piezoelectric ultrasonic instrumentation on titanium discs: a microscopy and trace elemental analysis in vitro study. Int J Dent Hyg.

[3] Sripathi Rao B, Bhat V (2015). Dental implants: a boon to dentistry. Arch Med Health Sci.

[4] Sidambe AT (2014). Biocompatibility of advanced manufactured titanium implants: a review. Materials (Basel).

[5] Hassan A, Taib H, Abas N, Zhen DP, Hanafi NN (2017). Evaluation of different hygiene instruments on titanium implant abutment. Int Med J.

[6] Hawker S, Payne S, Kerr C, Hardey M, Powell J (2002). Appraising the evidence: reviewing disparate data systematically. Qual Health Res.

[7] Speelman JA, Collaert B, Klinge B (1992). Evaluation of different methods to clean titanium abutments. A scanning electron microscopic study. Clin Oral Implants Res.

[8] Kawashima H, Sato S, Kishida M, Yagi H, Matsumoto K, Ito K (2007). Treatment of titanium dental implants with three piezoelectric ultrasonic scalers: an in vivo study. J Periodontol.

[9] Biazussi BR, Perrotti V, D'Arcangelo C, Elias CN, Bianchini MA, Tumedei M, de Vasconcellos DK (2019). Evaluation of the effect of air polishing with different abrasive powders on the roughness of implant abutment surface: an in vitro study. J Oral Implantol.

[10] Mengel R, Buns CE, Mengel C, Flores-de-Jacoby L (1998). An in vitro study of the treatment of implant surfaces with different instruments. Int J Oral Maxillofac Implants.

[11] Sinjari B, D'Addazio G, Bozzi M, Celletti R, Traini T, Mavriqi L, Caputi S (2018). Comparison of a novel ultrasonic scaler tip vs. conventional design on a titanium surface. Materials (Basel).

[12] Sahrmann P, Winkler S, Gubler A, Attin T (2021). Assessment of implant surface and instrument insert changes due to instrumentation with different tips for ultrasonic-driven debridement. BMC Oral Health.

[13] Unursaikhan O, Lee JS, Cha JK (2012). Comparative evaluation of roughness of titanium surfaces treated by different hygiene instruments. J Periodontal Implant Sci.

[14] Duarte PM, Reis AF, de Freitas PM, Ota-Tsuzuki C (2009). Bacterial adhesion on smooth and rough titanium surfaces after treatment with different instruments. J Periodontol.

[15] Kim JH, Herr Y, Chung JH, Shin SI, Kwon YH (2011). The effect of erbium-doped: yttrium, aluminium and garnet laser irradiation on the surface microstructure and roughness of double acid-etched implants. J Periodontal Implant Sci.

[16] Kim HK, Park SY, Son K, Kim YG, Yu WJ, Lee KB, Lee JM (2020). Alterations in surface roughness and chemical characteristics of sandblasted and acid-etched titanium implants after irradiation with different diode lasers. Appl Sci (Switzerland).

[17] Scarano A, Lorusso F, Inchingolo F, Postiglione F, Petrini M (2020). The effects of erbium-doped yttrium aluminum garnet laser (Er: YAG) irradiation on sandblasted and acid-etched (SLA) titanium, an in vitro study. Materials (Basel).

[18] Homiak AW, Cook PA, DeBoer J (1992). Effect of hygiene instrumentation on titanium abutments: a scanning electron microscopy study. J Prosthet Dent.

[19] Bahari ZF, Awang RA, Hassan A (2021). Effects of different prophylaxis procedures on titanium implant fixture: a scanning electron microscopy study. J Int Dent Med Res.

[20] Matsuyama T, Aoki A, Oda S, Yoneyama T, Ishikawa I (2003). Effects of the Er:YAG laser irradiation on titanium implant materials and contaminated implant abutment surfaces. J Clin Laser Med Surg.

[21] Azizi B, Azizi V, Peeva‐Petreska M, Vuletic M, Susic M, Gabric D (2019). Antimicrobial photodynamic therapy and light‐activated disinfection efficacy in decontaminating titanium implant surfaces: in vitro study. Clin Oral Impl Res.

[22] Saffarpour A, Nozari A, Fekrazad R, Saffarpour A, Heibati MN, Iranparvar K (2018). Microstructural evaluation of contaminated implant surface treated by laser, photodynamic therapy, and chlorhexidine 2 percent. Int J Oral Maxillofac Implants.

[23] Faramarzi M, Sadighi M, Mirhashemi S (2018). Evaluation of surface changes of dental implants after irradiation with diode laser beams with different energies: A SEM study. J Adv Periodontol Implant Dent.

[24] Gonçalves F, Zanetti AL, Zanetti RV, Martelli FS, Avila-Campos MJ, Tomazinho LF, Granjeiro JM (2010). Effectiveness of 980-mm diode and 1064-nm extra-long-pulse neodymium-doped yttrium aluminum garnet lasers in implant disinfection. Photomed Laser Surg.

[25] Kanathila H, Pangi A, Benakatti V, Patil S (2018). Maintenance of dental implants: a way to long term success: a review. Int J Appl Dent Sci.

[26] Gulati M, Govila V, Anand V, Anand B (2014). Implant maintenance: a clinical update. Int Sch Res Notices.

[27] Rapley JW, Swan RH, Hallmon WW, Mills MP (1990). The surface characteristics produced by various oral hygiene instruments and materials on titanium implant abutments. Int J Oral Maxillofac Implants.

[28] Augthun M, Tinschert J, Huber A (1998). In vitro studies on the effect of cleaning methods on different implant surfaces. J Periodontol.

[29] Louropoulou A, Slot DE, Van der Weijden FA (2012). Titanium surface alterations following the use of different mechanical instruments: a systematic review. Clin Oral Implants Res.

[30] Brookshire FV, Nagy WW, Dhuru VB, Ziebert GJ, Chada S (1997). The qualitative effects of various types of hygiene instrumentation on commercially pure titanium and titanium alloy implant abutments: an in vitro and scanning electron microscope study. J Prosth Dent.

[31] (2012). Safety guidelines for the laser removal of dental calculus. Laser Ther.

